# Time Spent on Social Media and Risk of Depression in Adolescents: A Dose–Response Meta-Analysis

**DOI:** 10.3390/ijerph19095164

**Published:** 2022-04-24

**Authors:** Mingli Liu, Kimberly E. Kamper-DeMarco, Jie Zhang, Jia Xiao, Daifeng Dong, Peng Xue

**Affiliations:** 1 Department of Psychology, Hunan University of Science and Technology, Xiangtan 411201, China; xiaojia201995@163.com; 2 Department of Psychology, State University of New York College at Buffalo, Buffalo, NY 14222, USA; kamperke@buffalostate.edu; 3 Department of Sociology, Central University of Finance and Economics, Beijing 102206, China; zhangj@buffalostate.edu; 4 Department of Sociology, State University of New York College at Buffalo, Buffalo, NY 14222, USA; 5 Medical Psychological Center, The Second Xiangya Hospital of Central South University, Changsha 410011, China; daifengdong@csu.edu.cn; 6 China National Clinical Research Center for Mental Disorders (Xiangya), Changsha 410011, China; 7 College of Psychology, Liaoning Normal University, Dalian 116082, China; xuepeng4869@163.com

**Keywords:** social media use, depression, adolescents, meta-analysis, dose–response

## Abstract

Adolescent depression is a worldwide public health concern and has contributed to significant socioeconomic burden. Investigating the association between time spent on social media (TSSM) and depression may provide guidance toward the prevention and intervention of adolescent depression. However, related literature reported mixed findings in terms of the relationship between TSSM and depression in adolescents. Hence, we conducted a comprehensive dose–response meta-analysis to clarify this issue. We conducted a systematic title/abstract and topic search of the relative terms in Web of Science, PubMed, PsycINFO databases through 9 January 2022. Odd ratios (ORs) were used to examine the pooled effect size of the association between TSSM and risk of depression. Dose–response analysis was evaluated by a generalized least squares trend estimation. Twenty-one cross-sectional studies and five longitudinal studies including a total of 55,340 participants were included. Overall, more TSSM was significantly associated with a higher risk of depression symptoms (OR = 1.60, 95%CI: 1.45 to 1.75) with high heterogeneity (Q_(29)_ = 105.9, *p* < 0.001; I^2^ = 72.6%). The association was stronger for adolescent girls (OR = 1.72, 95%CI: 1.41 to 2.09) than boys (OR = 1.20, 95%CI: 1.05 to 1.37). Five studies with seven reports were included in dose–response analysis. There was a linear dose–response association of TSSM and risk of depression. The risk of depression increased by 13% (OR = 1.13, 95%CI: 1.09 to 1.17, *p* < 0.001) for each hour increase in social media use in adolescents. TSSM is associated with depression in a linear dose–response and gender-specific manner, which suggests the need for better monitoring of adolescent social media use. However, motivation, content, and engagement on and exposure to social media use may also be important contributing factors, making it necessary to interpret the current findings with caution. Therefore, further research is required to clarify not only the causal link between TSSM and depression by randomized control studies but also the influence of other factors, such as active vs. passive social media use or different types of engagement or environments in which social media is used.

## 1. Introduction

Social media, also known as social networking, are internet-based interactive platforms where individuals and communities share and communicate [1,2]. In society today, children and adolescents grow up having both in-person and virtual social connections through social media (e.g., Facebook, Instagram, and WeChat) [3]. This continually emerging internet-based social communication has greatly expanded adolescents’ ability to make friends worldwide and makes it possible to connect with others anytime and anywhere. However, there is an ongoing debate about whether social media use is harmful to mental health or not [4,5], with some prior findings highlighting the psychological risk, especially depression, associated with excessive time spent on social medial (TSSM) in adolescence [6], while other studies report that there are only circumstantial correlations between TSSM and psychological problems [7]. Therefore, whether TSSM is associated with adolescents’ mental health concerns is still unclear. Notably, an increase in depression has emerged in adolescence, particularly in adolescent girls, over the past ten years [4]. Social media use has also been increasing rapidly at the same time [3,6]. Thus, it is necessary to obtain insight into the association between TSSM and depression in adolescents.

Several theories may explain the inconsistent findings regarding TSSM and depression in adolescents. Based on both the uses and gratifications theory [8] and self-determination theory [9], adolescents may gain a sense of belonging [10,11] and increased self-esteem [12,13] through social media, which is then associated with lower levels of depression. In support of these theories, the association of TSSM and depression in adolescents would follow a U-shaped curve as a previous work suggested [14]. One study determined that the lowest risk of depression in adolescence was found when individuals use approximately 1 h of screen time per day compared with the no screen time group. Some work has also demonstrated the benefits of social media use on depression risk, while other studies have reported very small or null correlations between the two [7,15,16,17,18]. Currently, there are more studies reporting a significant positive association between TSSM and depression in adolescents [6,19,20,21,22], with a recent study supported a J-shaped curve between TSSM and depression [5]. The displacement hypothesis [23] may help to explain the dark side of excessive TSSM on depression. According to this hypothesis, TSSM may replace time for productive and/or active activities, such as physical activity or face-to-face interpersonal communication, thereby influencing adolescents’ overall mental health, including depressive symptoms. Meanwhile, the strain theory may also explain the link between excessive TSSM and higher depression. Strains are usually caused by negative life events [24]. In a heavy involvement in TSSM, adolescents may experience more value strain, aspiration strain, and deprivation strain. All those strains are more likely to lead to depression [25]. Undoubtedly, another way to account for the high correlation between the two variables is to say that depressed adolescents may be more likely to indulge in social media to kill time [26] because depressed individuals have a negative cognitive bias, which may impair adolescents’ self-regulation and result in excessive social media use. Finally, these contradictory findings regarding social media use and adolescent depression may also be related to the different methodologies that these studies used, such as the use of different populations and measurements.

Another noteworthy variable is gender. Most studies reported mixed-gender results about social media use and depression. Recently, researchers found that associations between TSSM and depression are different in boys and girls, with TSSM only associated with depression for girls [19,27]. This is in line with research demonstrating that girls use much more social media and place more importance on the closeness of their interpersonal relationships than boys [5], which may then lead to more relational aggression, fear of missing out on social media, and depression [28,29]. It should be noted that one study did find that boys’ TSSM was also associated with depression; however, there was a stronger correlation for girls [6]. It is important to note that the relationship between TSSM and depression across gender is far more complicated than is outlined above. Numerous other factors may also affect these associations, such as active or passive use of social media [30], motivations for use [31], or environments to which adolescents are exposed [32].

To clarify the association between social media use and risk of depression in adolescents, several reviews have qualitatively summarized this association in children and adolescents [3,30,33,34]; however, these reviews often lack quantitative assessments. Relatedly, in these reviews, social media use is often measured more broadly, including other information, such as frequency, purpose, and investment or addiction of social media use, instead of specifically TSSM. One prior meta-analysis [35] pooled the correlation of social media use (not specifically time-based) and depression in adolescents from 11 studies and found a small but statistically significant positive correlation with high heterogeneity. In another meta-analysis, social media use was measured using both TSSM and frequency of social media use, and similar findings were reported [36]. However, responses regarding frequency of social media use were most commonly measured on a scale from “never” to “almost every day”, which have little variability because most adolescents use social media every day [5]. Similarly, neither meta-analysis explored sources of heterogeneity, and there was no pooled estimation of the relation between TSSM and risk of depression for adolescents, which may be important for providing evidence-based guidelines regarding TSSM. Thus far, there has been no meta-analysis that has examined a dose–response association between TSSM and risk of depression. In sum, it is clear that a more comprehensive meta-analysis is needed to quantify the dose–response association between TSSM and the risk of depression in adolescents.

The purpose of the current study is to summarize evidence related to the association between TSSM and depression in adolescents by pooling the risk of depression with TSSM for adolescents, quantifying a dose–response association, and exploring the heterogeneity of the included studies. Based on displacement theory, we hypothesize that more TSSM will be associated with a higher risk of depression in adolescents, with a linear dose–response association. Moderation by gender will also be included as an exploratory hypothesis. However, because of its non-experimental nature, no causal inferences can be drawn in the current study.

## 2. Methods

### 2.1. Search Strategy

This study was conducted according to the PRISMA guidelines [37] (see Table S1). Electronic databases, including Web of Science, PubMed, and PsycINFO, were searched systematically using title/abstract, and topic (through 9 January 2022) with no publication type or language restriction. To determine “social media” related search terms, a stratified searching strategy was adopted. Firstly, we searched the PubMed database using the most general terms of social media, such as “social media”, “digital media”, “social networking”, “SNS”, and “screen media”. Next, we screened all study titles and hundreds of abstracts, ending up with 56 different terms. Based on using frequency and generality, we divided them into two categories: general social media-related terms and specific social media-related terms. All general social media terms were included in the final search. However, some rarely used specific social media-related terms, such as “Digg”, and “Edmodo”, were deleted. Finally, three sets of medical subject terms (MeSH) and their combinations were used in the search, including “social media”, “social network*”, “SNS”, “digital media”, “screen media”, “online media”, “internet media”, “collaborative filtering site*”, “media sharing site*”, “Mashups”, “Facebook”, “Twitter”, “Instagram”, “YouTube”, “Snapchat”, “LinkedIn”, “WhatsApp”, “Pinterest”, “Blog”, “Wiki”, “Tumblr”, “Myspace”, “Google+”, “Reddit”, “WeChat”, “QQ”, “WordPress”, “Telegram”, “Flickr”, “Skype”, “Vine”, “Tweeting”, “podcasts”, “Tik Tok”, “Sermo”, “Google Groups”, “Forum and Blog”, “Second Life”; “depress*”; “adolescen*”, “juvenile*”, “teenager*”, “high school student*”, “middle school student*”, “children”. The asterisk indicates that the search was inclusive of larger words that contained the word or word fragment. Additionally, references of retrieved articles were screened.

### 2.2. Inclusion and Exclusion Criteria

**Figure 1:**
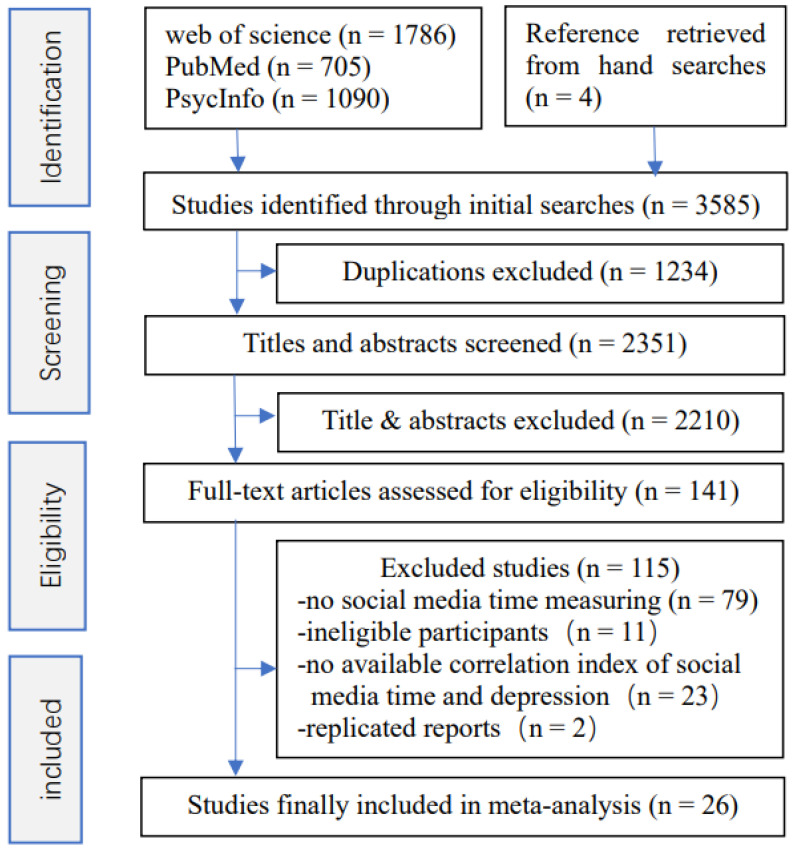
Flow chart of article screening process.

Studies were included if the following criteria were fulfilled: they were an observational study; they reported complete correlation indices of TSSM with depression which could be subsequently converted into an odds ratio (OR) with 95%CI; and average participant age was between 10 and 19 years old. Articles not meeting the inclusion criteria were excluded. Studies were also excluded if they reported mixed screen time, such as time spent playing internet games or watching online videos, as this would cause the measure of TSSM to be unclear. Authors were contacted if data were missing. Only one study was included if multiple articles reported the same research. The screening of titles/abstracts and topic and subsequent full-text assessment were performed independently by two authors (J.X. and P.X.). When the two authors made different decisions, they discussed the full text and determine its eligibility for inclusion together. If the two authors still disagreed, a third author (M.L.) helped to resolve the disagreement. Figure 1 displays the screening process.

### 2.3. Data Extraction

All related data (i.e., the first author’s name, published year, country, study objective, study design, participants’ gender and age, sample size, number of cases (for dose–response analyses), the detailed measure of TSSM and depression, and the correlation index of TSSM with depression) of eligible studies were extracted using EpiData V.3.1 and Excel by two investigators. For the quality assessment, we referenced the Meta-analysis of Observational Studies in Epidemiology (MOOSE) [38] and the Strengthening the Reporting of Observational Studies in Epidemiology (STROBE) [39] guidelines. Study quality was rated on a scale with a maximum of 8 points based on the following criteria: appropriate selection of participants (1 point); proper measures of TSSM (2 points) and depression (2 points); appropriate methods to deal with the design issues (1 point); appropriate handling of confounds (1 point) and proper statistical methods (1 point).

### 2.4. Statistical Analyses

Pooled data were expressed as ORs with 95%CIs. Studies that provided effect sizes stratified by gender were treated as two separate reports. For the studies reporting correlation coefficients, we converted the correlation coefficients to ORs with 95%CIs [40]. For one study using 1–3 h/day as the reference category for TSSM, we recalculated the ORs and 95%CIs using the no TSSM group as the reference category [40]. For studies that provided multiple categories’ effect sizes, we combined the corresponding estimates using the Excel RRs proposed by Hamling et al. [41]. The Q statistic was used to evaluate the heterogeneity among studies, and it was quantified by I^2^. Low, moderate, and high heterogeneity were indicated by the 25%, 50%, and 75% values of I^2^, respectively. If I^2^ < 50%, a fixed-effects model was used to estimate the pooled OR and corresponding 95%CI; otherwise, a random-effects model was administrated. To assess the sources of heterogeneity, we performed several subgroup analyses including gender, geographical regions, the measure of TSSM and depression, and sample size. In addition, sensitivity analyses were conducted to test the robustness of the results. Furthermore, tunnel plot asymmetry was used to detect publication bias of the included studies in this meta-analysis, and then Begg’s and Egger’s tests were performed to measure the publication bias.

A specific dose–response analysis was conducted to further estimate the association between TSSM and risk of depression. For the studies that did not report the median or mean of each category, the dose was calculated as the midpoint of the lower and upper boundaries in each group; for the open-ended lower or upper group, the boundary was assumed as the same as the closest group. Both non-linear and linear associations between TSSM and depression were tested. The potential non-linear dose–response relationship between TSSM and depression was estimated using a restricted cubic spline model with three knots of the TSSM distribution. Significance was then tested by setting the second spline coefficient equal to zero. A random-effects model was conducted to examine the trend because of the high heterogeneity among the studies. The dose–response coefficients and corresponding 95%CIs were calculated using a generalized least squares regression. The significance level was set at *p* < 0.05. All statistical analyses of this study were performed with STATA V12 software (Stata Corp, College Station, TX, USA).

## 3. Results

### 3.1. Characteristics of the Included Studies

According to the inclusion and exclusion criteria, 30 reports from a total of 26 studies, including a total of 55,340 participants, were included in the final analyses (see Figure 1). The characteristics of the included studies are summarized in Table 1. Twenty-one studies [6,15,16,17,18,19,20,21,22,27,42,43,44,45,46,47,48,49,50,51] were cross-sectional, and five were longitudinal [52,53,54,55,56]. Of note, one longitudinal study conducted by Coyne et al. [27] was actually a cross-sectional design for the relationship between TSSM and depression because they reported eight cross-sectional correlations based on data collected from each wave in eight years (2009 to 2017). We incorporated the seventh wave data, which was conducted in the most recent year and which also met the age criteria (19 years). For another four-wave longitudinal study [56], the authors reported a general between-persons and a within-persons regression association with means and standard deviations in the first wave and the last wave. We converted the data into ORs with 95%CIs using the general between-persons correlation for the four waves, with means and standard deviations in the last wave. Across all studies, sample size varied widely from 85 to 11,423 participants. Five studies [6,19,27,49,51] analyzed gender groups separately. However, we combined the total effect size for one study [51] that reported gender-specific results because the sample sizes of the single genders were too small. The mean age of all participants ranged from 11 to 19 years. Three studies reported the age range of the participants with no exact mean ages provided [21,42,47]. Eleven studies were conducted in Europe [6,16,18,19,21,43,47,49,52,53], nine in North America [27,31,42,44,46,48,50,51,56], four in Asia [15,17,45,55], one in Brazil [20], and one in Australia [22]. For the measure of TSSM, most of the studies (22 of 26) used total TSSM while the other four studies [31,43,46,53] used time spent on specific social media platforms, such as Facebook or Instagram. Meanwhile, several questionnaires, including the Center for Epidemiological Studies-Depression scale [57] (CESD, 11 studies), the Short version of the Mood and Feelings Questionnaire (SMFQ, 7 studies) [58], the Beck Depression Inventory [59] (BDI, 2 studies), the Patient Health Questionnaire-9 [60] (PHQ9, 3 studies), the Children’s Depression Inventory [61] (CDI, 1 study), the Brief Symptom Inventory [62] (BSI, 3 studies), the Hospital Anxiety and Depression Scale [63] (HADS, 1 study), the scale of the Original Symptom Checklist-Depression dimension [64] (OSCD, 1study), and one question asking “how often you felt depressed” [54] were used to measure depressive symptoms across all included studies. The quality score of all included studies ranged from 3 to 7, with 19 studies obtaining a score of greater than 5 (see Table S2).

### 3.2. Associations between TSSM and Depression Risk

**Figure 2:**
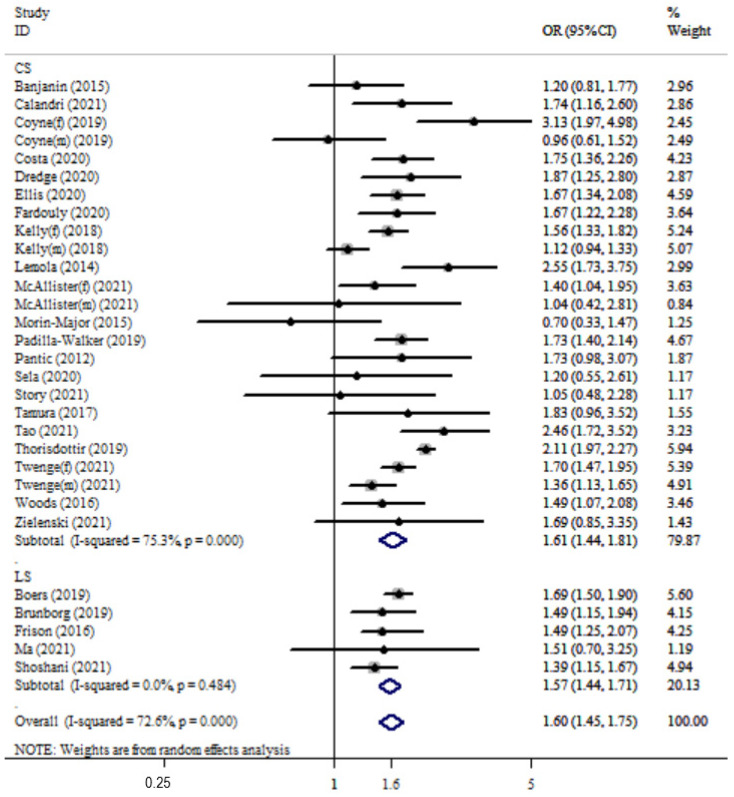
Forest plot of the association between time spent on social media (hours/day) and risk of depression in adolescents by study design. OR of depression for higher daily time using social media compared with reference groups and corresponding 95%CI. CS, cross-sectional; LS, longitudinal; f, female; m, male.

The overall pooled OR was 1.59 (95%CI: 1.44 to 1.77; *p* < 0.001) with high heterogeneity (Q_(27)_ = 105.9, *p* < 0.001; I^2^ = 72.6%). The combined OR was 1.61 (95%CI: 1.44 to 1.81) with high heterogeneity (Q_(24)_ = 97.25, I^2^ = 75.3%) for cross-sectional studies and 1.57 (95%CI: 1.44 to 1.71) with almost zero heterogeneity (Q_(4)_ = 3.46, I^2^ = 0%) for longitudinal studies (see Figure 2).

### 3.3. Subgroup and Sensitivity Analyses

Subgroup analyses show that the association between TSSM and risk of depression was moderated by gender and the measure of depressive symptoms (see Table 2).

For sensitivity analyses, no single study influenced the result significantly when studies were individually omitted (see Figure S1). There was also no significant change in the results when studies where another effect size was converted to an OR were excluded from analysis (the pooled OR was 1.47, 95%CI: 1.29 to 1.67, *p* < 0.001; I^2^ = 54.4%).

### 3.4. Publication Bias

**Figure 3:**
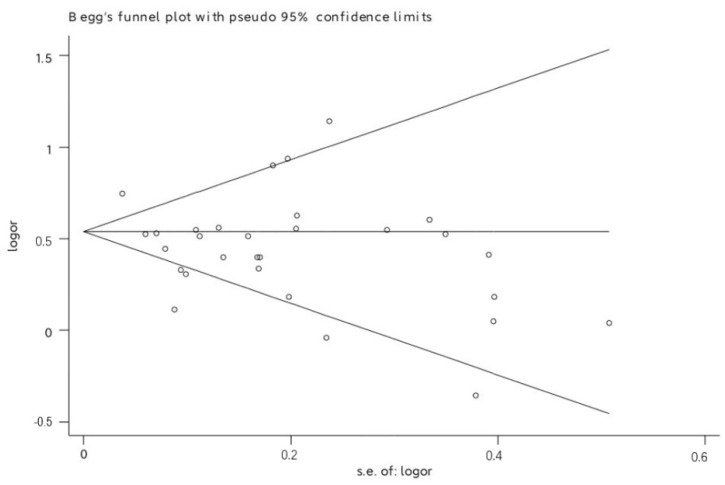
Funnel plot of publication bias.

Begg’s test did not show significant publication bias (*p* = 0.986) (see Figure 3), and Egger’s linear regression test suggested a mildly significant publication bias (*p* = 0.039). However, no trimming was needed to be performed when the nonparametric trim-and-fill method was used, demonstrating the reliability of the findings (see Figure S2).

### 3.5. Dose–Response Association between TSSM and Risk of Depression

**Figure 4:**
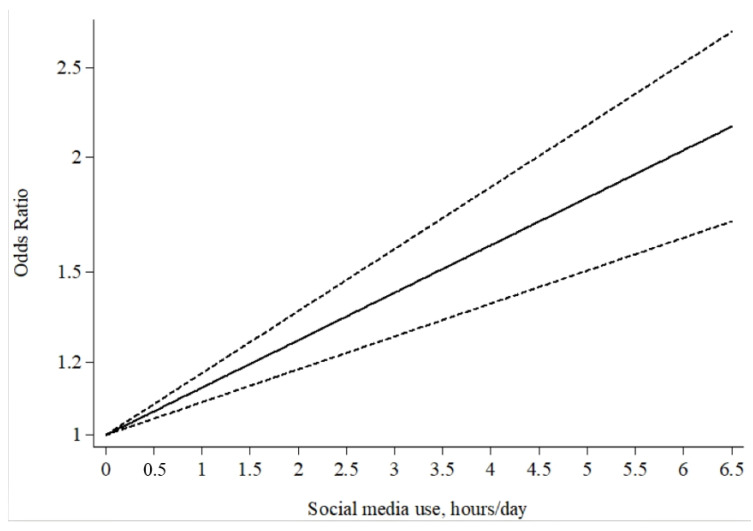
The generalized least squares trend estimated dose–response of time spent on social media and risk of depression in adolescents. Time of social media use was modelled with a restricted cubic spline in a two-stage random-effects dose–response model. The ORs are plotted on the log scale. Dashed lines represent the 95%CIs for the spline model. No social media use served as the referent category.

Five studies [6,17,19,20,54] (seven reports) were included for the dose–response analysis. The results showed a total linear association between TSSM and risk of depression (*p* = 0.888 for non-linearity, *p* < 0.001 for linearity; see Figure 4) with high heterogeneity between studies (Q = 70.33, *p* < 0.001). The risk of depression increased by 13% (OR = 1.13, 95%CI: 1.09 to 1.17, *p* < 0.001) for each hour increase in social media use in adolescents. For samples in which gender was examined separately, there were linear associations between TSSM and depression for both girls and boys (*p* = 0.720 for non-linearity). Specifically, the risk of depression increased by 13% (OR = 1.13, 95%CI: 1.08 to 1.16, *p* < 0.001) for girls and by 9% (OR = 1.09, 95%CI: 1.03 to 1.15, *p* = 0.002) for boys for each hour increase in social media use in adolescents.

## 4. Discussion

The present comprehensive meta-analysis investigated the association between TSSM and the risk of depression in adolescents. Our findings reveal that adolescents with higher daily TSSM had a 59.6% increase in terms of the risk of depression when compared with the reference group. Furthermore, the risk of depression increased by 13% for each hour increase in social media use, and these associations were stronger for adolescent girls than boys; however, boys still demonstrated a significant increase in depression risk. The findings are consistent with the WHO guidelines, which recommend limiting daily screen time for adolescents [65] and which are in agreement with the detrimental effect of high levels of social media use for adolescents suggested by some previous studies [4,5]. Moreover, the linear dose–response analysis of the current study demonstrated that with an increase in hours spent on social media each day, the risk of adolescent depression increased linearly. Therefore, it can be inferred that excessive TSSM may be a strong risk factor for adolescents’ depression. Consistently, Twenge et al. [6] suggested that excessive TSSM (>5 h) was associated with a more than 2-fold risk of depression (OR = 2.31, 95%CI: 1.98 to 2.70 for girls; OR = 2.05, 95%CI: 1.59 to 2.64 for boys), after controlling for relevant covariates such as age, family income, ethnicity, and presence of biological father; a similar risk of depression was found in the highest TSSM group (after recalculating using the 0 h/day category as the reference category) in girls (>5 h) in the study conducted by Kelly et al. [19]. Of note, although the included studies in the current meta-analysis have controlled for most relevant covariates (e.g., age, gender, family income, etc.), some other covariates (e.g., physical activity, which was shown to be a protective factor for adolescent’ depression) could also influence the results [66,67]. The current findings still need further support by future studies controlling all related covariates.

One notable finding of the current meta-analysis is the significant difference in the pooled estimate between boys and girls. Generally, a significant positive association between TSSM and risk of depression emerges in both girls and boys; however, this pattern is much larger for girls. This finding is consistent with previous studies regarding the association between adolescent social media use and risk of depression [6,68], but it is inconsistent with a longitudinal study examining media exposure (e.g., television, videocassettes, video games, and radio) and risk of depression [69]. In the aforementioned study, the authors found that a lower risk of depression was associated with more total media exposure for teenage girls. Social media use specifically, which was not assessed in this longitudinal study, could underlie these inconsistent results. Social media provides individuals multiple ways of seeking and maintaining social bonds [22,29,45]. Adolescents who fear of missing out social communication hope to continually stay connected with their peers and to stay updated on others’ states [70]. Currently, adolescent girls spend much more time on social media than boys [5,6,19,20,32], which may be attributed to the tendency among girls to emphasize close, intimate friendships [28,71]. Therefore, girls are more likely to experience fear of missing out [72] or being harassed [19,20] on social media, which has been associated with risk of depression [28,29]. Thus, it is understandable that previous studies may not have detected gender differences when examining total screen time (including video/computer games, computer/internet use, and television) and risk of depression. Studies stratified by media or screen category and gender are needed to clarify this question. Higher TSSM was associated with a higher risk of depression in the current meta-analysis across both younger and older adolescence. This seems inconsistent with a previous review [14] in which a significant screen time–depression risk was detected only in younger adolescents (<14 years). One possible reason is that some studies included in the current meta-analysis had a range of both younger and older adolescents [20,27,42,45,46,47,53]. For example, the study conducted by Woods et al. included participants ranging in age from 11 to 17 years [47]. Some social media platforms have an age limit for creating social media pages (e.g., 13 years of age for Facebook), which may also influence the results. Many studies with no stratification by age precluded us from clarifying the issue yet highlight an important area of future study. The included studies in this meta-analysis also used multiple different questionnaires measuring depressive symptoms. Interestingly, depression measurement type played a significant moderating role in the association between TSSM and risk of depression. Specifically, the two studies [15,16] in which depression was measured using the BDI demonstrated no significant correlation. The way in which this measure assessed depression may be an important factor to consider. In fact, previous work has asserted that the BDI is a good measure for identifying major depressive disorder [73]; thus, it may not be as accurate when examining a non-clinical population. Similarly, the sample sizes of the two studies were relatively small (*n* = 85/160), which may be non-comparable and may have caused a misleading correlation. Further studies with a larger sample size are needed to clarify differences in depressive symptoms related to specific measurement scales.

The current meta-analysis comprehensively quantified the dose–response association between TSSM and the risk of depression in adolescents. The large sample size allowed the meta-analysis of dose–response associations between TSSM, ranging from low to high duration, and risk of depression. This study also provided more precise results with smaller confidence intervals than in the previous original studies. International and national guidelines or strategies [74,75] promoting limited screen time for children and adolescents are supported by the current study. Although the implication of the study is not explicit because research is still evolving in this field, our findings reinforce TSSM limitations for adolescents in a gender-specific manner, particularly for girls, noting that the risk of depression increased linearly with an increase in daily hour of social media use. These results are important for adolescents and their parents or other caregivers because they clarify the potential risk of unlimited time on social media, and in turn, prompt them to take steps to promote positive adolescent health and development. Although research concerning links between TSSM and depression in adolescents has given rise to the development of media use policies, particularly regarding smart phone use, most school policies permit their students to use phones during non-instructional times in a school day, such as during recess and lunch [76]. Our findings provide more evidence for school policymakers, as well as national and international public health policymakers, for developing guidelines for appropriate social media use and consumption to reduce depressive risk for adolescents. On the other side, considering the effects of digital technology on the field [77], future study on digital technology innovations is also required for better assisting in “co-care” monitoring adolescents’ media use.

There are also important limitations to this study, making it necessary to interpret the findings with caution. First, all included studies were observational, in which the results may be influenced by other potential covariates not yet considered. Hence, we cannot speak to causality in the interpretation of the results. Relatedly, adolescents who had higher depressive symptoms may have recall bias in which they tend to endorse excessive social media use more so than those who had fewer depressive symptoms. As a result, studies in which social media use is measured more objectively are needed in the future. Second, although the search strategies did not restrict language, English databases may lead to the omission of non-English articles as well as non-English terms around social media, which may have an important role in better understanding this association. Although we used a stratified search strategy, there are still various specific social media platforms and social media-related terms, especially those only used in specific countries or regions, that were not included. Third, distinct measurement scales of depressive symptoms and diagnostic criteria for depression could increase variability across included studies. More studies with consistent instruments and diagnostic criteria for depression are needed to support the current findings. Relatedly, understanding the differences in association within a clinical vs. non-clinical population may be important for better understanding for whom TSSM may have a more harmful effect. Most of the participants of eligible studies were collected from Europe and North America, which may limit the generalization of the current findings. Therefore, further investigations from other cultures, particularly focusing on developing countries/regions, are needed to replicate the current findings.

## 5. Conclusions

Our findings provide evidence that more TSSM is associated with a higher risk of depression in adolescence in a linear dose–response manner, especially for teenage girls. Therefore, prevention efforts targeting a better understanding of the effects of TSSM, particularly for adolescent girls, may be a key component to lessen the risk of depression as social media continues in its global popularity. However, other variables, such as motivation, different platforms, and exposure to social media use may influence this association, making it necessary to interpret the findings with caution. Future research using randomized control studies is required to clarify the causal link between TSSM and depression, as well as the different effects of how adolescents use social media and the environments in which they use it.

## Figures and Tables

**Table 1:** The Characteristics of the included studies.

Study	Design	Main Study Objective	Country; Sample Size (Female)	Age (Years)	Measure of Time Spent on Social Media	Depression Measure
Banjanin et al. 2015	CS	Investigated the potential relationship between internet addiction and depression in adolescents.	Serbia; 336 (66%)	18	Self-report daily time spent on social networking; Response: self-administered open answer	CESD
Boers et al. 2019	LS	Repeatedly measured the association between screen time and depression.	Canada; 3826 (47%)	12.7–15.7 Grade 7–11	Self-report how much time per day they spend on social networking sites; Response: 0–30 min, 30 min–1.5 h, 1.5 h–2.5 h, ≥3.5 h	BSI
Brunborg et al. 2019	LS	Examined association between time spent on social media and depression, conduct problems, and drinking.	Norway; 763 (55%)	15.22	Self-report daily hours spent on social media; Response: <1 to >15 in hourly increments	PHQ9
Calandri et al. 2021	LS	Investigated the relationships between social media use and depressive symptoms.	Italy; 336 (48%)	13.0 (13–15)	Self-report daily hrs spent on communicating online with friends through social networks; Response: 0, 1, 2, ≥3	CESD
Costa et al. 2020	CS	Examined the associations between self-reported and accelerometer-measured movement behaviors and depressive symptoms.	Brazil; 610 (52%)	16.30 (14–18)	Self-report daily hours spent on social media; Response: <2, 2–4, ≥4	CESD
Coyne et al. 2019	CS	Examined the association between time spent using social media and depression and anxiety at the intra-individual level.	USA; 500 (52%)	13–20	Self-report daily hours on social media; Response: 1 (0) to 9 (>8)	CESD
Dredge et al. 2020	CS	Examined the association between online gaming and social media use frequency, depression, and other mental health.	China; 320 (47%)	13.98 (12–17)	Self-report daily time spent on social media; Response: 1 (0) to 9 (>8)	PHQ9
Ellis et al. 2020	CS	Examined the relationships between psychological adjustment and stress and the initial COVID-19 crisis.	Canada; 1054 (76%)	16.68 (14–18)	Self-report daily time spent using social media platforms; Response: <10 min, 10–30 min, 31–60 min, 1–2 h, 2–3 h, 3–5 h, 5–10 h, to more than 10 h	BSI
Fardouly et al. 2020	CS	Investigated differences between preadolescent users and non-users of various social media platforms on mental health.	Australia; 528 (269)	11.19	Self-report daily time spent on social media platform; Response: 0 (0), 1 (<5 min), 2 (5–15 min), 3 (15–30) min, 4 (30 min–1 h), 5 (1–2 h), 6 (2–4 h), 7 (4–6 h), 8 (6–8 h), 9 (8–10 h), 10 (10–12 h or more).	SMFQ
Frison et al. 2016	LS	Examined the relationships between peer victimization on Facebook, depressive symptoms, and life satisfaction.	Belgium; 1621 (51%)	14.76 (12–19)	Self-report daily hours spent on Facebook; Response: 0 (0), 1 (0.5), 2 (0.5–1), 3 (1–1.5), 4 (1.5–2), 5 (2–2.5), 6 (2.5–3), 7 (3–4), 8 (4–5), 9 (>5), 10 (always logged in and available for interaction)	CESD
Kelly et al. 2018	CS	Assessed association between social media use and adolescents’ depressive symptoms.	UK; 10,904 (50%)	14.30	Self-report daily hours spent on social media; Response: 0, <1, 1–3, 3–5, ≥5	SMFQ
Lemola et al. 2014	CS	Sought a better understand the interplay between sleep, depressive symptoms, and electronic media use at night	Switzerland; 362 (45%)	14.82 (12–17)	Self-report daily duration spent online on Facebook; Response: self-administered open answer	CESD
Ma et al. 2021	LS	Examined how time spent on types of screen use was associated with depressive symptoms.	Sweden; 3556 (51%)	8 grades	Self-report daily hours spent on social media; Response: >2, 2, 1, <1, 0	Question of how often felt depressed
McAllister et al. 2021	CS	Compared associations across specific screen media activities and examined associations with self-harm behaviors.	UK; 4243 (55%)	13.75 (13–15)	Self-report time diary on one weekday and one weekend day from 4:00 am one day to 4:00 am the next day; for each 10 min time slot	SMFQ
Morin-Major et al. 2015	CS	Explored the associations between Facebook and basal levels of cortisol among adolescents.	Canada; 94 (53%)	14.50 (12–17)	Self-report weekly time spent on Facebook; Response (hours): 1 (<1), 2 (2–5), 3 (6–10), 4 (11–15), 5 (16–20), 6 (>21)	CDI
Padilla-Walker et al. 2019	CS	Explored the links between parental media monitoring and adolescents’ internalizing symptoms.	USA; 1155 (51%)	10–20	Self-report daily time spent on social media; Response: 1 (none), 2 (less than 30 min), 3 (31–60 min), 4 (1–2 h), 5 (2–3 h), 6 (3–4 h), 7 (5–6 h), 8 (7–8 h), and 9 (≥9 h)	CESD
Pantic et al. 2012	CS	Investigated the relationship between social networking and depression in adolescent.	Serbia; 160 (68%)	18.02	Self-report daily time spent on social networking sites; Response: self-administered open answer	BDI
Sela et al. 2020	CS	Tested the association between family environment and excessive internet use among adolescents.	Israel; 85 (41%)	14.04 (12–16)	Objectively measure time logged in various social medias on the smartphone for 14 days; Response: average time per day spent on social media.	BDI
Shoshani et al. 2021	LS	Examined the influence of the COVID-19 pandemic on children and adolescents’ mental health and well-being, and potential risk and protective moderators.	Israel; 1537 (52%)	13.97	Self-report daily hours spent on social media; Response: 0, <1, 1, 2, 3, 4, 5, 6, ≥7.	BSI
Story 2021	CS	Assessed the link between the time spent on social networking sites and depression among 9th and 10th grade high school students.	USA; 85 (56.5%)	14.88 (14–16)	Self-report the number of times and the number of min they spent on SNS daily. Response: sum of the min was divided by the sum of the times	PHQ
Tamura et al. 2017	CS	Investigated the relationship between mobile phone use and insomnia and depression in adolescents.	Japan; 295 (41%)	16.20 (15–19)	Self-report daily time spent on social networking sites; Response (min): 0, <30, 30–60, 60–120, ≥120	CESD
Tao et al. 2021	CS	Assessed the relationships among social media use, individual and vicarious social media discrimination, and mental health.	USA; 407 (82%)	16.47 (15–18)	Self-report Total time spent on social media per week; Response: multiple days/week by h/day	CESD
Thorisdottir et al. 2019	CS	Documented the prevalence of social media use and investigate the relationship of both active and passive social media use to anxiety and depressed mood.	Iceland; 10,563 (50%)	14–16	Self-report daily hours on social media; Response: 1 (0) to 8 (≥6)	OSCD
Twenge et al. 2021	CS	Examined associations between different types of screen activities and mental health.	UK; 11,423 (50%)	13.77 (13–15)	Self-report hours spent on social networking or messaging sites on a normal weekday during term time; Response: <0.5, 0.5–0.99, 1–1.99, 2–2.99, 3–4.99, 5–6.99, ≥7	SMFQ
Woods et al. 2016	CS	Examined how social media use related to sleep quality, self-esteem, anxiety and depression.	UK; 467	11–17	Self-report daily hours spent on social media; Response: 1 (<1) to 6 (>6)	HADS
Zielenski et al. 2021	CS	Examined the relationship between Instagram use, social comparison, and depressive symptoms.	USA; 110 (56%)	12–18	Self-report daily hours spent on Instagram; Response:<1 h; 1–2 h; 2–3 h; 3–4 h; 4–5 h; >5 h	CESD

Note: CS, cross-sectional study; LS, longitudinal study; CESD, the Center for Epidemiological Studies-Depression scale; SMFQ, the short version of the Mood and Feelings Questionnaire; BDI, the Beck Depression Inventory; PHQ9, the Patient Health Questionnaire-9; CDI, the Children’s Depression Inventory; BSI, the Brief Symptom Inventory; HADS, the Hospital Anxiety and Depression Scale; OSCD, the scale of the Original Symptom Checklist-Depression dimension.

**Table 2:** Moderation analyses for time spent on social media–depression risk association.

Variables	K	OR	95%CI	Z	Heterogeneity Test		
					I^2^(%)	Q_w_	*p*-Value
Gender, Q_b(2)_ = 40.44 ***							
Boys	4	1.20	1.05–1.37	2.62 *	8.9	3.29	0.349
Girls	4	1.72	1.41–2.09	5.38 ***	66.8	9.03	0.029
Mixed	22	1.67	1.52–1.84	10.27 ***	60.8	53.14	0.001
Age, Q_b(2)_ = 9.28 **							
<14	10	1.54	1.34–1.79	5.85 ***	54.9	19.96	0.018
>14	17	1.61	1.41–1.84	7.10 ***	79	76.11	<0.001
Mixed	3	1.66	1.40–1.97	5.73 ***	0	0.55	0.758
Regions, Q_b(3)_ = 4.13							
Europe	14	1.54	1.33–1.79	5.74 ***	82.8	75.58	<0.001
North America	10	1.68	1.41–1.99	5.88 ***	62.1	23.73	0.005
Asia	4	1.47	1.25–1.73	5.38 ***	0	2.41	0.491
Others	2	1.72	1.41–2.09	4.73 ***	0	0.05	0.820
Measure of Time Spent on Social Media, Q_b(1)_ = 0.23							
Total	26	1.	1.45–1.76	9.39 ***	73.7	95.11	<0.001
Specific	4	1.56	1.01–2.40	1.99	71.6	10.56	0.014
Measure of Depression, Q_b(5)_ = 56.7 ***							
SMFQ	7	1.44	1.26–1.65	5.20 ***	62.3	15.92	0.014
CESD	11	1.77	1.48–2.10	6.39 ***	60	24.98	0.005
BDI	2	1.52	0.96–2.41	1.79	0	0.55	0.458
PHQ9	3	1.55	1.25–1.91	4.04 **	0	1.88	0.391
BSI	3	1.59	1.41–1.80	7.50 ***	36.2	3.14	0.208
Others	4	1.51	1.02–2.24	2.04 *	76.4	12.73	0.005
Sample Sizes, Q_b(1)_ = 0.35							
>1000	13	1.55	1.37–1.76	6.88 ***	83.3	33.5	0.006
<1000	17	1.65	1.42–1.92	6.54 ***	52.3	72.050	<0.001

Note: SMFQ, short version of the Mood and Feelings Questionnaire; CESD, the Center for Epidemiological Studies-Depression scale; BDI, the Beck Depression Inventory, PHQ9, the Patient Health Questionnaire-9; BSI, Brief Symptom Inventory; * *p* < 0.05; ** *p* < 0.01; *** *p* < 0.001.

## Data Availability

The raw data supporting the conclusions of this article will be made available by the authors, without undue reservation, by the corresponding author.

## Supplementary Materials

The following supporting information can be downloaded at: https://www.mdpi.com/article/10.3390/ijerph19095164/s1. Table S1: The PRISMA checklist of this meta-analysis; Table S2: The risk of bias for included studies; Figure S1: The sensitivity analyses by omitting the included studies one by one; Figure S2: The metatrim analysis by nonparametric trim-and-fill method.
