# Supplementary material for: Time Spent on Social Media and Risk of Depression in Adolescents: A Dose–Response Meta-Analysis
Source: Int J Environ Res Public Health. 2022 Apr 24;19(9):5164. doi: 10.3390/ijerph19095164 (PMC9103874; doi:10.3390/ijerph19095164)
Supplement: Supplementary file 1 [file ijerph-19-05164-s001.zip › Figure S2. Metatrim Analysis.pdf]

Meta-analysis Metatrim

Method	Pooled Est	95% CI Lower	95% CI Upper	Asymptotic z_value	Asymptotic p_value	No. of studies
Fixed	0.539	0.499	0.579	26.182	0.000	30
Random	0.468	0.375	0.562	9.821	0.000	

Test for heterogeneity: Q= 103.662 on 29 degrees of freedom (p= 0.000)
Moment-based estimate of between studies variance = 0.036

Trimming estimator: Linear
Meta-analysis type: Fixed-effects model

iteration	estimate	Tn	# to trim	diff
1	0.539	133	0	465
2	0.539	133	0	0

Note: no trimming performed; data unchanged

Filled
Meta-analysis (exponential form)

Method	Pooled Est	95% CI Lower	95% CI Upper	Asymptotic z_value	Asymptotic p_value	No. of studies
Fixed	1.714	1.647	1.785	26.182	0.000	30
Random	1.597	1.455	1.754	9.821	0.000	

Test for heterogeneity: Q= 103.662 on 29 degrees of freedom (p= 0.000)
Moment-based estimate of between studies variance = 0.036
