# Supplementary material for: Time Spent on Social Media and Risk of Depression in Adolescents: A Dose–Response Meta-Analysis
Source: Int J Environ Res Public Health. 2022 Apr 24;19(9):5164. doi: 10.3390/ijerph19095164 (PMC9103874; doi:10.3390/ijerph19095164)
Supplement: Supplementary file 1 [file ijerph-19-05164-s001.zip › Table S2 Risk of bias for included studies.pdf]

Table S2. Risk of bias for included studies

Study	design[†]	population	measurement method for social media time	measurement method for depression	control confounding	methods to deal with any design-specific issues[‡]	statistical methods	Quality score
Banjanin, 2015	CS	*	**	-	-	*	-	5
Boer et al. 2019	LS	*	*	**	*	*	*	7
Brunborg, 2019	LS	*	**	**	*	-	-	7
Calandri, 2021	CS	-	**	**	-	-	*	5
Costa, 2020	CS	*	*	*	*	*	*	6
Coyne, 2019	CS	*	**	**	*	*	-	7
Dredge, 2020	CS	*	**	**	*	-	-	6
Ellis, 2020	CS	*	**	**	*	-	-	6
Fardouly,2020	CS	*	**	**	*	-	-	6
Frison, 2016	LS	*	**	**	*	*	-	7
Kelly, 2018	CS	*	**	*-	*	-	*	6
Lemola, 2014	CS	*	**	**	*	-	-	6
Ma, 2021	LS	*	**	*	*	-	*	6
McAllister, 2021	CS	*	**	*	*	*	*	7
Morin-Major, 2015	CS	-	**	**	*	-	-	5
Padilla-Walker, 2019	CS	*	**	**	*	*	-	7
Pantic, 2012	CS	-	**	*-	-	*	-	4
Sela, 2020	CS	-	**	**	*	*	-	6
Shoshani, 2021	LS	*	**	**	-	-	*	6
Story, 2021	CS	-	*	**	-	-	-	3
Tamura, 2017	CS	-	*-	**	-	*	*	5
Tao, 2021	CS	*	**	**	-	-	*	6

Thorisdottir, 2019	CS	*	**	**	*	*	-	7
Twenge, 2021	CS	*	**	**	*	–	*	7
Woods, 2016	CS	*	**	**	*	–	–	6
Zielenski, 2021	CS	-	*	**	*	–	*	5

† CS, cross-sectional study; LS, longitudinal study; LC, longitudinal cohort study; ‡ appropriate methods outlined to deal with any design-specific issues including: recall bias, interviewer bias for cross-sectional studies; and biased loss to follow-up but excluding confounding for longitudinal studies; *, 1 point; **, 2 points
