# Supplementary figures and images for: Time Spent on Social Media and Risk of Depression in Adolescents: A Dose–Response Meta-Analysis
Source: Int J Environ Res Public Health. 2022 Apr 24;19(9):5164. doi: 10.3390/ijerph19095164 (PMC9103874; doi:10.3390/ijerph19095164)

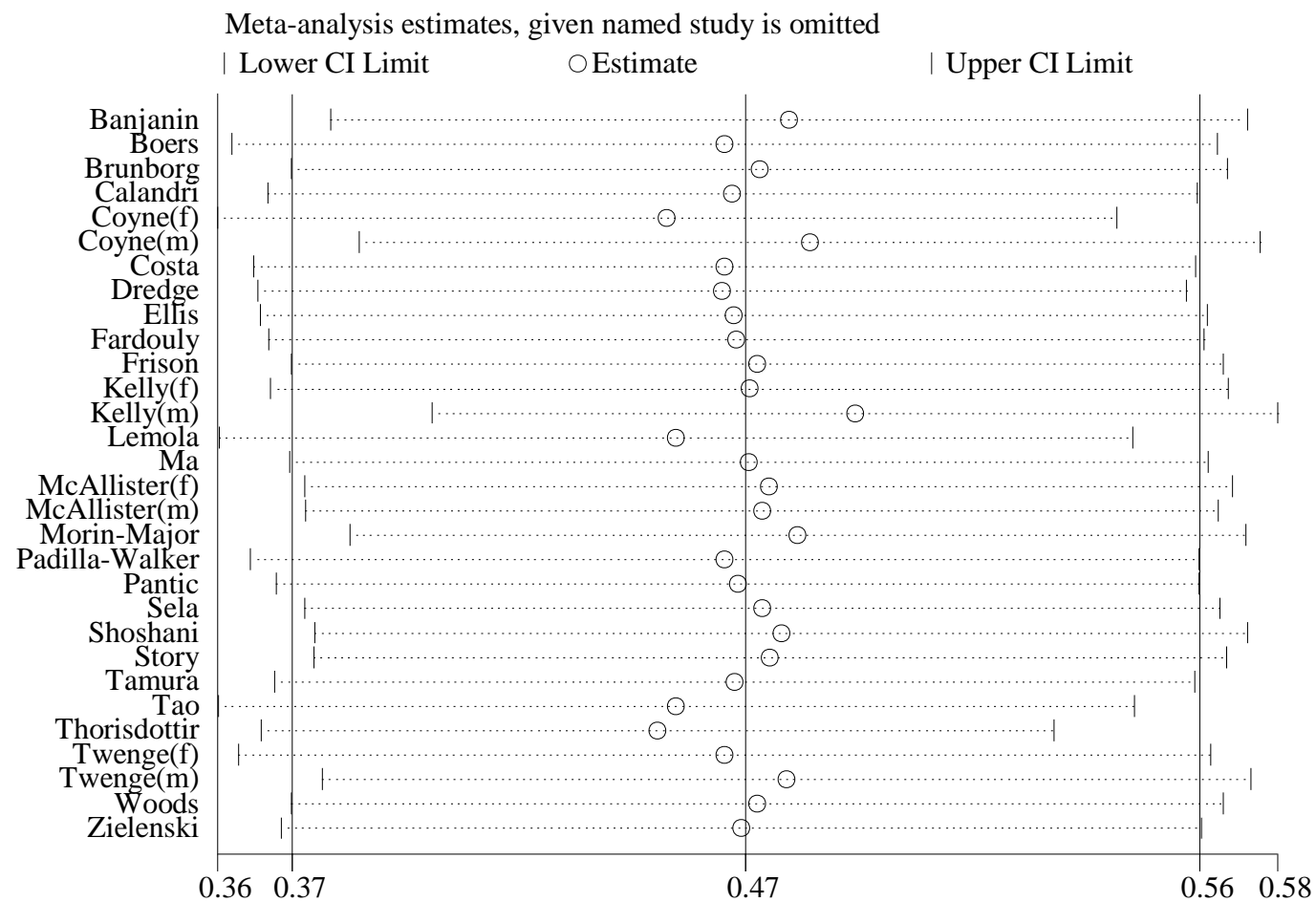

Supplement: Supplementary file 1 [file ijerph-19-05164-s001.zip › Figure S1 Sensitivity analyses for included studies.pdf]
